# Unlocking the Potential of Assisted Hatching in Assisted Reproductive Technology: A Narrative Review

**DOI:** 10.7759/cureus.60736

**Published:** 2024-05-21

**Authors:** Suhas Deotalu, Akash More, Priti Karadbhajne, Kamlesh Chaudhari

**Affiliations:** 1 Clinical Embryology, Datta Meghe Institute of Higher Education and Research, Wardha, IND; 2 Obstetrics and Gynaecology, Datta Meghe Institute of Higher Education and Research, Wardha, IND

**Keywords:** zona pellucida, embryo quality, fertility treatment, embryo hatching, implantation rate

## Abstract

The present study is set in the broad field of assisted reproductive technologies (ARTs) and examines various procedures under assisted hatching (AH). It also reviews their effects on implantation success rates. The primary emphasis is on explaining who has benefited and how many have benefited from these interventions. The most important factor determining the success rate of ART is implantation. To increase these rates, we use AH in our clinics to enhance each embryo’s chances at life and substantially improve overall results. This comprehensive review includes various approaches, such as chemical-based measures (such as applying Tyrode’s solution) and mechanical techniques (such as zona drilling and partial zona dissection). The individual techniques are carefully scrutinized, considering their mechanical detailing, methods of applying therapeutic effects, and the appropriateness of matching present social circumstances.

The review begins by analyzing the basic nature of AH as a medium for embryo implantation and then focuses on how this detailed view reveals the advantages and drawbacks of various methods. Moreover, the articles discuss improvements in AH technology and many of the most modern technological developments that can help fine-tune ART issues. A major problem with these methodologies is that they involve serious risks and legal complications. However, a broad assessment of these topics allows us to understand their impact on fertility treatments.

This review is written as a guidebook for physicians and researchers working in reproductive medicine. It compiles all current knowledge, providing literature to build on successes that will make breakthroughs possible in ART. Indeed, this is a valuable reference for guiding us in navigating complex AH procedures. It advances ARTs step by step toward perfection.

## Introduction and background

Over the last quarter of a century, there has been massive progress in assisted reproductive technology (ART), which is transforming modern fertility treatment and opening up new possibilities for people who are not childless. Assisted hatching (AH) has undoubtedly become a significant aspect of ART that people have noticed and are constantly perfecting. AH is the delicate procedure of making an opening in the protective outer layer of embryo tissue known as the zona pellucida to improve the chances for implantation during in vitro fertilization (IVF) and other assisted reproductive procedures [1,2]. In fact, the idea of AH grew out of observations suggesting that some embryos (originating from older women, for instance, or those subjected to cryopreservation) had a thicker or more impermeable zona pellucida. While the zona is essential in protecting the developing embryo, it can occasionally create obstacles during implantation. With this realisation, it was only natural that researchers and clinicians would seek methods to assist embryos in hatching from the zona, thereby increasing their odds of implantation into the uterine lining [3].

AH began with mechanical methods, in which a micropipette or a specific instrument was used to crudely puncture an opening in the zona pellucida [4]. However, this initial method had problems such as the fragile embryos could easily be damaged. Subsequent developments introduced chemical methods that used enzymatic or acid solutions to soften the zona selectively [5,6]. These chemical-assisted hatching methods were less invasive than mechanical methods and provided a more delicate and sensitive approach.

Laser technology then ushered in a new era of assisted hatching. The laser-assisted hatching (LAH) technique made previously unknown precision and control possible, allowing embryologists to create well-defined openings in the zona pellucida for the first time [2]. Various laser systems, including the infrared diode, neodymium-doped yttrium aluminum garnet (Nd:YAG), ruby, and erbium-doped yttrium aluminum garnet (Er:YAG) lasers, began to refine and optimize AH protocols.

In addition to being precise, these lasers allowed for real-time monitoring and adjustments during the procedure. The infrared diode laser, which operates in the non-ionizing infrared spectrum [7], offers a non-invasive and highly precise method for zona thinning. The Nd:YAG laser, with its high pulse energy, allows controlled and localized thinning with minimal impact on the embryo’s thermal structure [8]. Meanwhile, with minimal thermal effects, the Er:YAG laser allows better control during the hatching process. These laser systems became important components of the developing arsenal of AH techniques. Besides these laser systems, a newly introduced innovation is the non-contact diode laser [9]. Using the principle of precise but contactless operation, this revolutionary technique reduces the risk of mechanical stress or damage to the embryo. Combining precision with safety, a non-contact diode laser has demonstrated promise in zona thinning and offers embryologists an entirely new tool [10]. As AH techniques have introduced new ways of improving implantation rates and overall success in ART procedures, the field continues to face challenges and requires ongoing research. Safety remains a key area of concern, including the possible thermal effects caused by laser systems, the choice of chemical agents in chemical methods, and the impact on embryo health. Additionally, approaches that consider patient and embryo characteristics, as well as the nature and cause of infertility, are increasingly viewed as key to improving AH success rates [11].

In this comprehensive review, we will take a close look at the nuts and bolts of mechanical, chemical, and laser-assisted hatching techniques. We will examine how each type is performed, the results achieved, their safety profiles, and the advancements in these methods. By carefully evaluating the advantages and disadvantages of each method, we aim to provide a thorough overview of the current state of AH and its future prospects in ART.

## Review

Methodology

**Figure 1:**
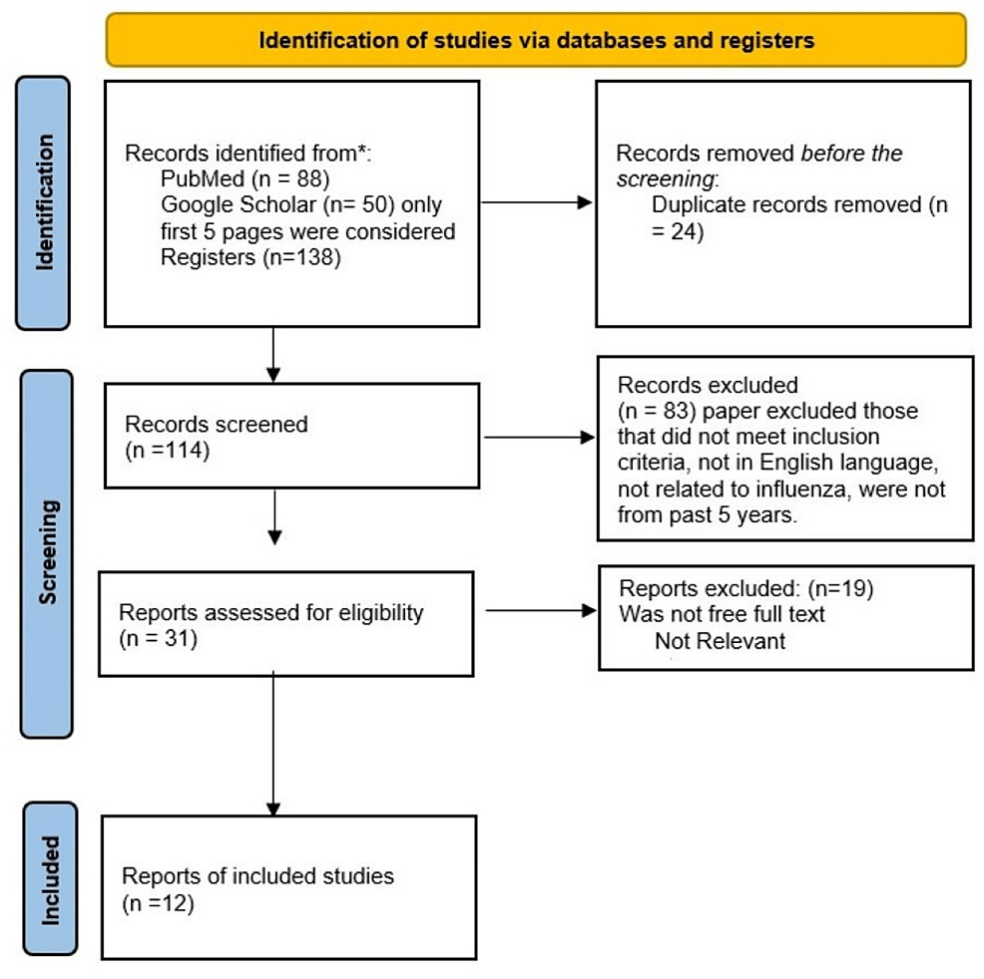
Flowchart of the article selection process.

This analysis systematically scrutinizes the extensive literature on AH techniques in ART. We utilized search terms such as “assisted hatching” and “in vitro fertilisation,” established criteria for including relevant studies in this review, thoroughly screened results for detailed analysis, and retrieved full-text articles for a comprehensive examination. Our data extraction process involved gathering pertinent information from specific studies, including study design and participant characteristics, AH methods, and relevant findings from reputable sources such as PubMed and Google Scholar. Quality standards were employed to evaluate the rigor of studies, considering factors such as sample size and potential bias. Its systematic nature enabled the identification of patterns and trends across multiple studies. After conducting the initial search, we identified 138 articles in the searched database. We then excluded 24 duplicate articles. In the initial screening, 83 were excluded because they were irrelevant to the topic. Following the full-text screening of the remaining 31 articles, we excluded 19 articles because they did not meet the inclusion criteria, only abstracts were available, and they were old studies. This left 12 articles for the final review (Figure 1).

The menu of AH techniques in ART resembles a veritable buffet platter. Each dish is designed to enhance the odds of successful embryo implantation during IVF. Here, we present a wide variety of methods for AH, both mechanical and chemical. In this field, some notable works have become guideposts for later discussions.

Mechanical assisted hatching

A traditional but basic technique in ART, mechanical AH is used to increase the embryo’s chances of implantation [11]. In this method, an opening is made in the zona pellucida (the protective outer layer of the embryo) either by using micropipettes or specialized tools. As it occurs during hatching, the micropipette is carefully guided either to thin or tear the zona and thereby aid the emergence of the embryo. Although mechanical AH offers a high degree of control over the technique, an expert embryologist is required to perform the procedure satisfactorily [12]. This approach is often associated with increased implantation rates, especially in cases where the zona pellucida appears thick and resistant [13]. Nevertheless, limitations such as the risk of damage to the embryo and the need for operator expertise indicate that AH techniques are still evolving [14], thus calling for comparison with newer approaches such as chemical and laser-assisted hatching. There are two types of mechanical AH, namely, zona drilling and partial zona dissection (PZD) [14].

Zona Drilling

The mechanical AH technique, zona drilling, involves cutting a small opening in a portion of the zona pellucida, the protective shell surrounding the embryo, using either a micropipette or a fine needle. With this precise approach, these cells can be closely cropped or punctured, presumably reducing the time spent in the zona pellucida and improving implantation potential [15]. This application of zona drilling has been particularly successful in cases where the zona pellucida appears abnormally thick or when failures to generate ongoing pregnancy after previous implantations are attributed to the quality of the zona [9]. Several studies have shown a significant improvement in implantation rate and pregnancy success following zona drilling procedures [16]. However, similar to other mechanical AH techniques, it requires careful thought and delicate handling to avoid any possible risk to the embryo [17]. Current research in the area is focused on fine-tuning zona drilling techniques and distinguishing them as a distinct aid for hatching.

Partial Zona Dissection

Partial zona pellucida dissection is a medical intervention that preserves the environment around the embryo by controlling the incision or opening in certain areas of the zona pellucida. This precision, often achieved using microblading or laser, allows the embryologist to carefully thin or excise a portion of the zona pellucida, speeding up the incubation process and potentially improving the embryo’s chances for successful implantation [18,19]. Studies have shown that PZDs can be particularly beneficial when the zona pellucida is thick or resistant, providing a good way to enhance embryo implantation potential [20]. Positive outcomes associated with PZD surgery include increased implantation and improved fertility [2]. However, as with other incubation procedures, careful evaluation of embryo health and embryologist expertise is important to mitigate risks [21]. Ongoing research aims to further refine PZD techniques and determine their effectiveness in various ARTs.

Chemical assisted hatching

One innovative technique used in ART is to use chemical agents to weaken or thin the zona pellucida, which surrounds the embryo. The purpose of this technique is to speed up the natural hatching process and aid implantation [22]. When the structure is modified, different enzymes, i.e, the proteolytic enzyme or dilute acid, can be used [23]. Chemical AH is one of the non-invasive methods commonly used when it is suspected that the zona may be thicker than ideal or resistant [24]. Several studies have demonstrated that chemical AH can improve the rate of implantation and increase pregnancy rates [25]. Nevertheless, this chemical approach requires a closer examination of the safety and effectiveness of each specific chemical applied. Of course, much research is also being done to improve protocols and enhance the measured impact or accuracy in taking measurements overall [26]. In clinical settings, chemically assisted fertilization is a field that requires specialized knowledge and understanding. Depending on the procedure, currently, there are two types of chemicals AH available, which are discussed below.

Enzymatic and Acidic Solutions in Zona Pellucida Thinning

In ART, chemical agents are divided into two categories, namely, enzymatic and acidic solutions. These serve to thin the zona pellucida during AH. The enzymatic approach employs proteolytic enzymes such as pronase, proteinase K, or trypsin, which selectively destroy certain proteins of the zona pellucida and result in gradual thinning [27,28]. However, acidic solutions such as hydrochloric or triode acids produce a chemical reaction that weakens the structure of the zona, making hatching easier [29,30]. Each technique attempts to achieve a selective opening in the zona pellucida, thus enhancing implantation potential for the embryo. Several studies have confirmed the value of enzymatic and acidic solutions in enhancing implantation rates, as well as the overall results for ART procedures [19,26]. However, the different features of each embryo, together with the abilities of respective embryologists and possible side effects from using chemical agents, must be considered before timely use in clinical practice.

Studying the role of proteolytic enzymes in processes relevant to hatching sheds light on some details about how exactly thinning occurs. Strategically, pronase or proteinase K is applied to the zona pellucida and positions itself around specific proteins within its structure. This enzymatic action ultimately leads to controlled digestion in which the zona is gradually thinned out, creating an opening through which developing embryos can escape [31,32]. The application of this method also provides embryologists with a more delicate means by which to adjust zona thinning for the individual quality characteristics of each embryo. According to the literature, when proteolytic enzymes are used in assisted reproduction, implantation rates improve, and doctors have more success with ART [26,33]. However, the selection of a specific kind and concentration, as well as the duration of exposure, must be weighed against the effectiveness it brings to zona thinning and its potential threat to embryo health. These ongoing efforts to refine the application of proteolytic enzymes and determine their safety and efficacy are intended to help couples achieve successful outcomes from ART.

Laser-assisted hatching

LAH can be considered a refined and high-precision technique within ART for artificially thinning the zona pellucida. Various laser systems, such as the infrared diode laser, Nd:YAG, and Er:YAG, are utilized for this purpose. This opening in the zona pellucida is precisely created, just large enough to aid hatching. With this technology, monitoring is done in real-time, and adjustments can be made accordingly. Compared to the current approach, it is also more precise while exerting reduced thermal stress on the embryo. There is convincing evidence that LAH can improve implantation rates and results in ART procedures [34,35]. However, careful consideration of the particular laser system, parameters, and operator experience is needed to optimize safety and efficacy. LAH has been further developing, with research progressing rapidly in this area, such as time-lapse imaging or the application of artificial intelligence for real-time monitoring and analysis. The technique used to treat infertility will become even more optimized.

Infrared Diode, Nd:YAG, and Er:YAG Laser Systems

In the field of LAH in the context of ART, three prominent laser systems have attracted attention due to their precision and effectiveness, i.e., the infrared diode laser, Nd:YAG laser, and Er:YAG laser. An infrared diode laser, emitting light in the infrared spectrum [36], provides a non-ionizing and minimally invasive form of zona pellucida thinning. The Nd:YAG laser has been shown to be efficient in puncturing openings that allow the embryo to hatch more easily and with less stressful exposure of its surface layer [37]. On the other hand, the Er:YAG laser, with its precision and infrared wave usually associated with low thermal impact, ensures safety through the LAH procedure [38]. Each of these laser systems, with its own characteristics, contributes to the progress in LAH by providing embryologists with a variety of ways and means for custom-made modifications as required on the zona pellucida. Current research is further clarifying the differences between different individual laser systems, as well as fine-tuning all application parameters to ensure that both precision and safety are maintained in general ART scenarios.

Precision and Control in Laser-Assisted Approaches

When using LAH techniques in ART, both precision and control are of prime importance. Laser systems, including the infrared diode laser, Nd:YAG, and Er:YAG, provide unrivaled accuracy in making a hole in the zona pellucida. The infrared diode laser works solely by the law of its wavelength and belongs to the infrared range. This is also a precision device that non-ionizes and thins the zona pellucida [36]. The Nd:YAG laser achieves controlled and localized thinning with low thermal stress on the embryo due to its high pulse energy [39]. The Er:YAG laser, with its high precision and low thermal impact, effectively monitors hatching [38]. With these laser systems, embryologists can make precise alterations to the zona pellucida with little risk of inadvertently damaging an embryo. Continuous improvement in laser parameters and the ongoing progression of technology improve the degree, stability, and control that can be achieved through laser-assisted procedures. This contributes to improved safety for various ART techniques.

Thermal Considerations and Safety Profiles

Thermal considerations and safety profiles are two important factors in the application of LAH to ART. Laser systems such as the infrared diode laser, Nd:YAG laser, and Er:YAG laser provide the necessary precision, creating an opening in the zona pellucida, but the thermal damage suffered by the embryo must also be considered [40,41]. The Nd:YAG laser has a high pulse energy and brings about some thermal effects, making safety an issue [42]. In contrast, the Er:YAG laser has low thermal impact, enhancing its safety profile and reducing potential harm in zona thinning [43]. Balancing the thermal aspects and safety profile is crucial: achieving accurate hatching results while preventing the embryo from suffering any unnecessary harm or discomfort. LAH techniques are continually improving in ART, with ongoing research seeking to reduce laser parameters and standardize procedures to enhance safety profiles.

Non-contact diode laser

In ART, the use of a non-contact diode laser has become a high-level tool, especially when coupled with LAH. This laser system is based on precision devoid of physical contact and provides a precise technique for making an opening in the zona pellucida during ART operations. Unlike contact methods, the non-contact diode laser reduces mechanical stress or damage to cells and provides greater safety [44]. This technology is especially well-suited for achieving precisely controlled thinning of the zona without a high thermal effect, thus preserving embryonic viability. It has been demonstrated that non-contact diode lasers can boost the success rate of implantation and overall successful embryo transfer with ART procedures while guaranteeing safety by implementing scientific principles [45,46]. With ART pursuing further development, the non-contact diode laser is being seen as a high-precision and safer laser hatching tool. Continuing research efforts are attempting to perfect the application parameters of this technology.

Implantation rates across different assisted hatching techniques

Seed production is an important parameter to evaluate the effectiveness of AH technology in the context of ART. This review examines the subtle differences in implantation rates observed with various AH methods and highlights their implications for successful IVF procedures. Studies using techniques such as zona pellucida drilling and partial zona pellucida dissection have shown variable but generally positive results in terms of implantation [21,26,47]. The introduction of chemical methods, especially the use of acidic Tyrode’s solution, has shown good results and affects the implantation rate by promoting embyro hatching [2]. In addition, recent technological advances, including the use of non-contact diode lasers, indicate the development of techniques that have the potential to further enhance growth by opening and keeping the zona pellucida open [48]. This comprehensive study highlights the various aspects of AH technology and its impact on different crops, providing insights to practitioners and researchers working to improve treatment outcomes when transitioning from ART.

Pregnancy and live birth rates in mechanical, chemical, and laser approaches

Evaluation of pregnancy and birth in different AH ART methods shows a nuanced picture of outcomes. Techniques such as zona pellucida drilling and partial zona pellucida dissection have been shown to be differently effective in improving pregnancy and survival [2,14,26]. Although these methods have been shown to be effective at implantation, their impact on the overall success of ART should be carefully evaluated. Medical procedures such as the use of acidic Tyrode’s solution show great difficulty in affecting pregnancy and birth. Chemical modification of the zona pellucida facilitates embryo hatching, but the translation of these effects into pregnancy and live birth requires further study [49,50]. Recent innovations, particularly the use of non-contact diode lasers, point to the forefront of technological development, with the potential to improve pregnancy and birth by the actual opening of the zona pellucida [1,51-53]. This research demonstrates the interplay between AH technology and reproductive outcomes as leading physicians and researchers seek strategies to improve fertility and childbearing in a dynamic ART environment.

## Conclusions

In summary, this comprehensive review covers the complex field of AH techniques in ART, including mechanical, chemical, and laser techniques. Building on the seminal work by previous researchers, the historical evolution of these technologies provides an important context for understanding their approaches. Mechanical methods (such as zona pellucida drilling and partial zona pellucida dissection) and chemical methods (such as applying acidic Tyrode’s solution) show differences between implantations and nuances in their results that effectively promote good outcomes. Pregnancy evaluation of live births with this method shows the interaction between selection and birth outcomes. Although mechanical methods provide good results, translating these results into pregnancy and birth requires careful evaluation. The use of chemical agents for modification of the zona pellucida presents both challenges and opportunities. Recent technological developments, especially non-contact diode lasers, point to a promising frontier with implications for the treatment of ART outcomes.

According to doctors and researchers conducting research in this field, the adaptation and foundation of the patient are important. This review serves as a compass to better understand the strengths and limitations inherent in various AH techniques. It provides insights into current practices and suggests avenues for future research. In the evolution of ART, this synthesis of knowledge is an important step for the continued development of reproductive support strategies, ultimately leading to improved pregnancy and patient survival.
